# A 1:1:1 co-crystal solvate comprising 2,2′-di­thiodi­benzoic acid, 2-chloro­benzoic acid and *N*,*N*-di­methyl­formamide: crystal structure, Hirshfeld surface analysis and computational study

**DOI:** 10.1107/S205698901900375X

**Published:** 2019-03-26

**Authors:** Sang Loon Tan, Edward R. T. Tiekink

**Affiliations:** a Research Centre for Crystalline Materials, School of Science and Technology, Sunway University, 47500 Bandar Sunway, Selangor Darul Ehsan, Malaysia

**Keywords:** crystal structure, di­thiodi­benzoic acid, chloro­benzoic acid, hydrogen bonding, Hirshfeld surface analysis, computational chemistry

## Abstract

The asymmetric unit of the three-component title compound, 2,2′-di­thiodi­benzoic acid–2-chloro­benzoic acid–*N*,*N*-di­methyl­formamide (1/1/1), C_14_H_10_O_4_S_2_·C_7_H_5_ClO_2_·C_3_H_7_NO, contains a mol­ecule each of 2,2′-di­thiodi­benzoic acid (DTBA), 2-chloro­benzoic acid (2CBA) and di­methyl­formamide (DMF). The DTBA mol­ecule is twisted [the C—S—S—C torsion angle is 88.37 (17)°] and each carb­oxy­lic group is slightly twisted from the benzene ring to which it is connected [CO_2_/C_6_ dihedral angles = 7.6 (3) and 12.5 (3)°]. A small twist is evident in the mol­ecule of 2CBA [CO_2_/C_6_ dihedral angle = 4.4 (4)°]. In the crystal, the three mol­ecules are connected by hydrogen bonds with the two carb­oxy­lic acid residues derived from DTBA and 2CBA forming a non-symmetric eight-membered {⋯HOCO}_2_ synthon, and the second carb­oxy­lic acid of DTBA linked to the DMF mol­ecule *via* a seven-membered {⋯HOCO⋯HCO} heterosynthon. The three-mol­ecule aggregates are connected into a supra­molecular chain along the *a* axis *via* DTBA-C—H⋯O(hydroxyl-2CBA), 2CBA-C—H⋯O(hydroxyl-DTBA) and DTBA-C—H⋯S(DTBA) inter­actions. Supra­molecular layers in the *ab* plane are formed as the chains are linked *via* DMF-C—H⋯S(DTBA) contacts, and these inter-digitate along the *c*-axis direction without specific points of contact between them. A Hirshfeld surface analysis points to additional but, weak contacts to stabilize the three-dimensional architecture: DTBA-C=O⋯H(phenyl-DTBA), 2CBA-Cl⋯H(phenyl-DTBA), as well as a π–π contact between the delocalized eight-membered {⋯HOC=O}_2_ carb­oxy­lic dimer and the phenyl ring of 2CBA. The latter was confirmed by electrostatic potential (ESP) mapping.

## Abstract 2

The three-component title compound contains a mol­ecule each of 2,2′-di­thiodi­benzoic acid (DTBA), 2-chloro­benzoic acid (2CBA) and di­methyl­formamide (DMF). The mol­ecules are connected *via* O—H⋯O hydrogen bonds between DTBA and 2CBA mol­ecules, and O—H⋯O hydrogen bonds between the second carb­oxy­lic acid of DTBA and the carbonyl group of the DMF mol­ecule.

## Chemical context   

Recent bibliographic reviews have highlighted the rich coord­ination chemistry based on ligands derived from 2-mercapto­benzoic acid (2-MBA) (Wehr-Candler & Henderson, 2016 ▸) and its 3- and 4-isomeric analogues (Tiekink & Henderson, 2017 ▸). By contrast, co-crystal formation with these mol­ecules is quite limited with the only co-crystal of an *n*-MBA mol­ecule being that formed between 2-MBA and its oxidation product 2,2′-di­thiodi­benzoic acid (DTBA) (Rowland *et al.*, 2011 ▸). One reason for the scarcity of co-crystals containing 2-MBA is the propensity for the acid to be oxidized, to generate DTBA, during co-crystallization experiments with bipyridyl-type mol­ecules (Broker & Tiekink, 2007 ▸) and with other carb­oxy­lic acids (Tan & Tiekink, 2019*a* ▸). Another, less common, outcome of crystallization experiments with 2-MBA is the sulfur extrusion product, 2,2′-thiodi­benzoic acid (Tan & Tiekink, 2018 ▸; Gorobet *et al.*, 2018 ▸). Herein, another unexpected product from a co-crystallization experiment involving 2-MBA is described. While the now anti­ci­pated coformer DTBA was observed after the co-cryst­allization of 2-MBA with 2-chloro­benzoic acid (2CBA), and recrystallization from a toluene/di­methyl­formamide solution (50:50 *v*/*v*), a solvent di­methyl­formamide mol­ecule was also found in the resultant co-crystal solvate. In this three-component crystal, one of the carb­oxy­lic acid groups of the DTBA mol­ecule forms hydrogen bonds to DMF rather than to 2CBA. Herein, the crystal and mol­ecular structures of the title co-crystal solvate are described along with an analysis of the calculated Hirshfeld surfaces and a computational chemistry study.
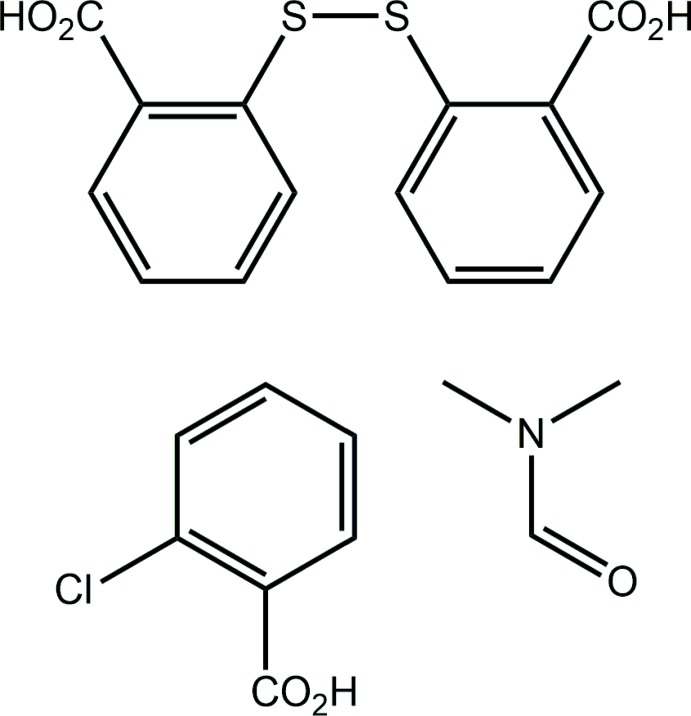


## Structural commentary   

**Figure 1:**
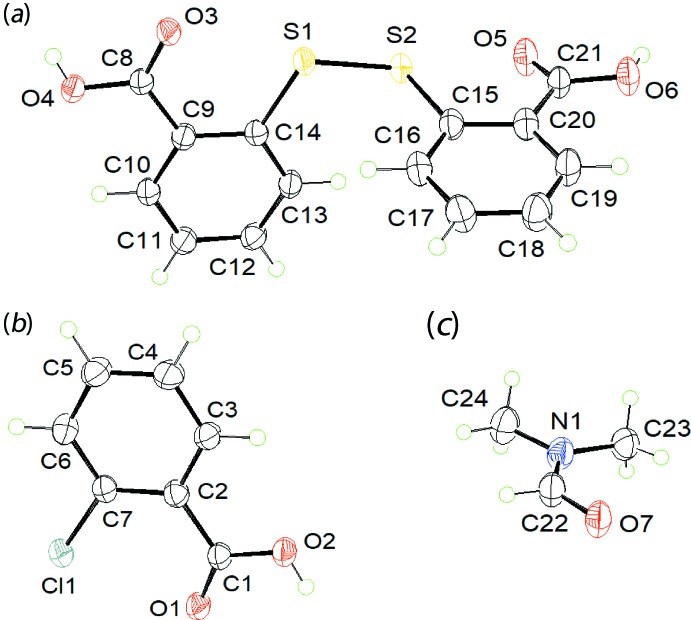
The mol­ecular structures of (*a*) 2,2′-di­thiodi­benzoic acid, (*b*) 2-chloro­benzoic acid and (*c*) di­methyl­formamide in (I), showing the atom-labelling scheme and displacement ellipsoids at the 50% probability level.

The title compound, (I), was isolated from the co-crystallization of 2-mercapto­benzoic acid and 2-chloro­benzoic acid prepared through solvent-assisted (methanol) grinding, followed by recrystallization from a toluene/di­methyl­formamide solution (50:50 *v*/*v*). The asymmetric unit comprises 2,2′-di­thiodi­benzoic acid (DTBA), 2-chloro­benzoic acid (2CBA) and a di­methyl­formamide (DMF) solvent mol­ecule in a stoichiometric 1:1:1 ratio, as illustrated in Fig. 1 ▸; each mol­ecule is in a general position.

As anti­cipated, crystallography reveals that the original 2-mercapto­benzoic acid underwent oxidation to yield a mol­ecule of DTBA, with the benzoic acid moieties being bridged through a di­sulfide bond [S1—S2 = 2.053 (1) Å]. The presence of carb­oxy­lic acid groups is confirmed by the disparity in the bond lengths for C8—O4, O3 [1.317 (4) and 1.229 (4) Å] and C21—O6, O5 [1.326 (4) and 1.209 (4) Å]. Both carb­oxy­lic acid groups (O3—C8—O4 and O5—C21—O6) are slightly twisted from the benzene rings (C9/C14 and C15/C20) to which they are bonded with the corresponding dihedral angles being 7.6 (3) and 12.5 (3)°, respectively. The C14—S1—S2—C15 torsion angle is 88.37 (17)°, indicating an almost orthogonal disposition between the benzene rings. The carbonyl-O3 and O5 atoms are oriented towards the di­sulfide-S1 and S2 atoms with S1⋯O3 and S2⋯O5 distances of 2.713 (2) and 2.661 (3) Å, respectively, and are indicative of hypervalent S←O inter­actions (Nakanishi *et al.*, 2007 ▸).

As for DTBA, the confirmation that 2CBA exists as a carb­oxy­lic acid is readily ascertained by the difference observed in the C1—O1, O2 bond lengths of 1.222 (4) and 1.320 (4), respectively. The carb­oxy­lic acid group is almost co-planar with the phenyl ring (C2–C7) as seen in the dihedral angle of 4.4 (4)° between their planes. Similarly, co-planarity is also noted between the chloride atom and benzene ring plane with the r.m.s deviation from the least-squares plane through the seven non-hydrogen atoms being 0.027 Å.

## Supra­molecular features   

**Figure 2:**
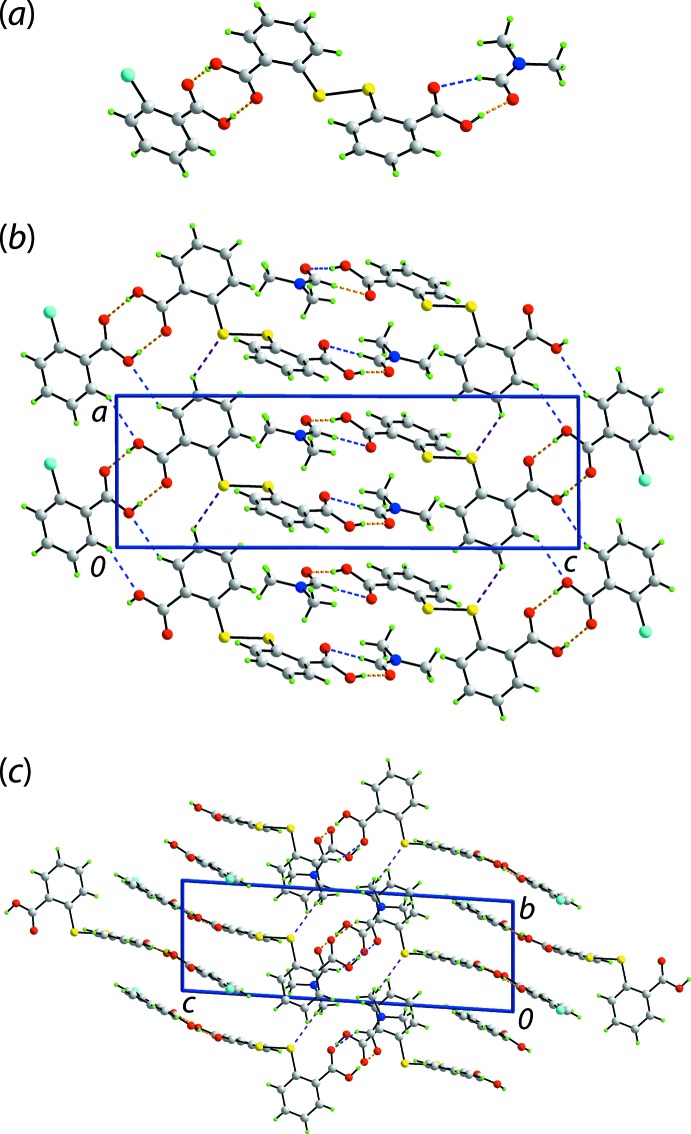
Mol­ecular packing in (I): (*a*) a view of the three-mol­ecule aggregate with the O—H⋯O hydrogen bonds and C—H⋯O inter­actions shown as orange and blue dashed lines, respectively, (*b*) supra­molecular chains aligned along the *a* axis with the C—H⋯S inter­actions shown as purple dashed lines and (*c*) a view of the unit-cell contents in perspective down the *a* axis.

The geometric parameters characterizing the inter­atomic contacts in the crystal of (I), as identified in *PLATON* (Spek, 2009 ▸), are given in Table 1 ▸. Some of the main contacts in the mol­ecular packing provide direct links between DTBA, 2CBA and DMF mol­ecules, in that hydrogen bonds are formed between one of the terminal carb­oxy­lic groups of DTBA and 2CBA, and between the other carb­oxy­lic acid terminus with the carbonyl group of DMF. The former inter­action leads to a classical, but non-symmetric eight-membered {⋯HOCO}_2_ homosynthon while the latter results in a seven-membered {⋯HOCO⋯HCO} heterosynthon when the C22—H22⋯O5 inter­action is taken into account, Fig. 2 ▸(*a*).

The resultant three-mol­ecule aggregates are connected by DTBA-C10—H10⋯O2(hydroxyl-2CBA) and 2CBA-C3—H3⋯O4(hydroxyl-DTBA) inter­actions to form a non-symmetric, ten-membered {⋯OCCCH}_2_ homosynthon, as well as discrete DTBA-C11—H11⋯S1(DTBA) inter­actions. These lead to a supra­molecular chain along the crystallographic *a* direction, as indicated in Fig. 2 ▸(*b*). Inter­actions between the chains leading to a layer in the *ab* plane occur through DMF-C24—H24*C*⋯S2(DTBA) contacts, Fig. 2 ▸(*c*). The layers inter-digitate along the *c*-axis direction with only weak contacts between them as detailed in the next section.

## Hirshfeld surface analysis   

To better understand the nature of the inter­molecular inter­actions in the crystal of (I), the individual mol­ecules comprising the asymmetric unit as well as the contents of the asymmetric unit were subjected to Hirshfeld surface analysis using *Crystal Explorer 17* (Turner *et al.*, 2017 ▸) and based on the procedures described in the literature (Tan *et al.*, 2019 ▸).

**Figure 3:**
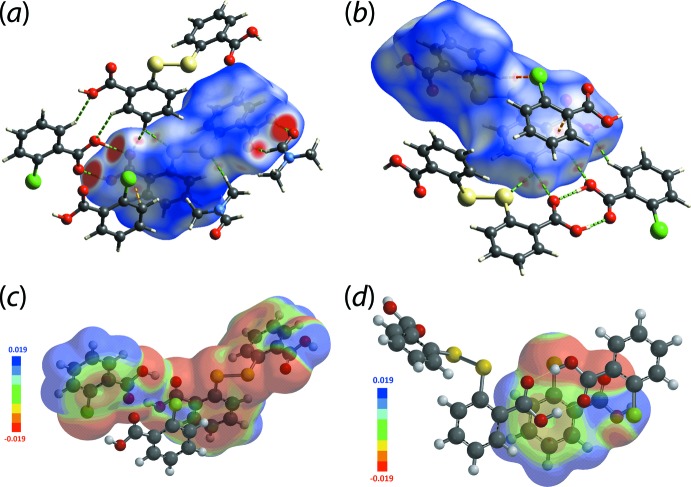
(*a*) and (*b*) Two views of the *d*_norm_ map of the DTBA mol­ecule within the range −0.274 to +0.862 arbitrary units, showing the short contacts highlighted as red spots with the intensity relative to the contact distances. Hydrogen bonds are indicated as green dashed lines and π–π contacts are highlighted as yellow dashed lines. ESPs map of (*c*) the DTBA dimer and (*d*) 2CBA, with the isosurface value scaled from −0.019 to +0.019 atomic units.

The *d*_norm_ maps of the respective mol­ecules in the aggregates are shown in Fig. 3 ▸. DTBA exhibits several intense red spots on the *d*_norm_ map signifying close contacts which origin­ate from DTBA-O—H⋯O(carbonyl-2CBA), DTBA-O—H⋯O(carbonyl-DMF), DTBA-C=O⋯H(hydroxyl-2CBA) and DTBA-C=O⋯H(DMF). Other red spots are observed through the *d*_norm_ map, albeit with relatively weak intensity. The contacts are consistent with those identified above except for some additional inter­actions such as DTBA-C=O⋯H(phenyl-DTBA), 2CBA-Cl⋯H(phenyl-DTBA) as well as a π–π contact between the delocalized eight-membered {⋯HOC=O}_2_ carb­oxy­lic dimer and the phenyl ring of 2CBA, Fig. 3 ▸(*b*). To validate the non-conventional π–π contact, the inter­acting mol­ecules were subjected to electrostatic potential (ESP) mapping using *Spartan’16* (Spartan’16, 2017 ▸) by treating the DTBA dimer as a single entity through a DFT-B3LYP/6-311+G(*d*,*p*) level of theory. The ESP mapping shows that the dimeric ring ranges from electropositive to neutral within the centre of the ring while the phenyl ring of 2CBA is mainly neutral indicating that the inter­action is mainly diffusive in nature, Fig. 3 ▸(*c*) and (*d*). As for the 2CBA and DMF mol­ecules, the corresponding *d*_norm_ maps (not shown) are reflective of their inter­actions with the DTBA mol­ecule.

**Figure 4:**
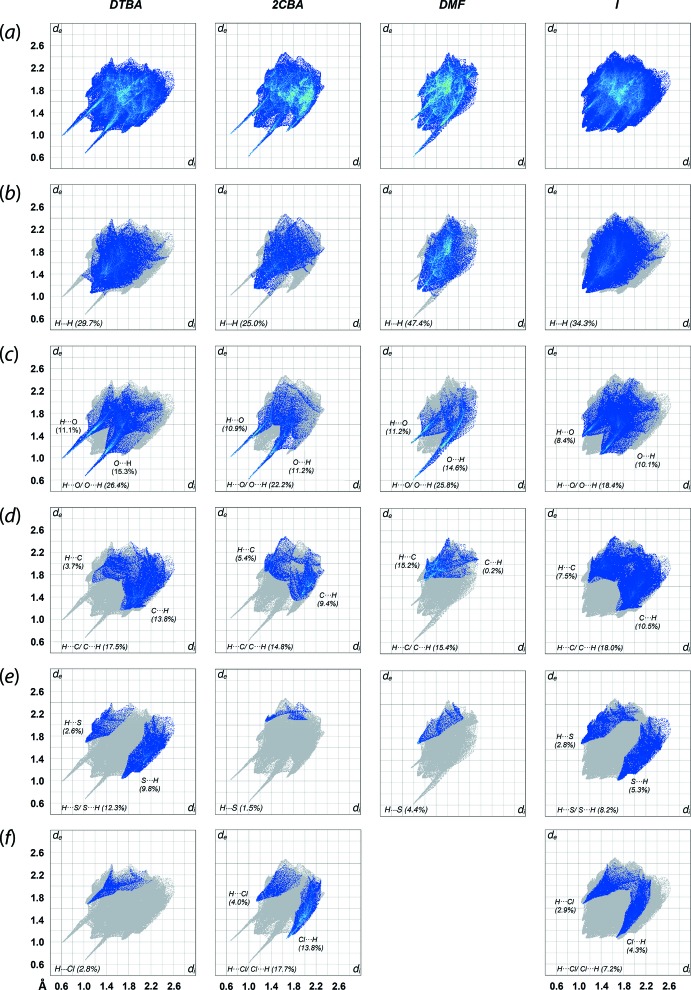
(*a*) The overall two-dimensional fingerprint plots for the DTBA, 2CBA and DMF mol­ecules and entire (I), and those delineated into (*b*) H⋯H, (*c*) H⋯O/ O⋯H, (*d*) H⋯C/ C⋯H, (*e*) H⋯S/ S⋯H and (*f*) H⋯Cl/ Cl⋯H contacts, with the percentage contribution being specified for each contact.

The two-dimensional fingerprint plots were generated to qu­antify the close contacts identified on the Hirshfeld surfaces. The overall fingerprint plot of (I) and the corresponding plots of the individual components are shown in Fig. 4 ▸. In general, (I) exhibits a shield-like profile in the overall fingerprint plot without any obvious spikes unlike the individual components. This indicates the discrete nature of the three-mol­ecule aggregate sustained by hydrogen bonding. Decomposition of the full fingerprint plots of (I) shows that the contacts are mainly dominated by H⋯H (34.3%; *d*_i_ + *d*_e_ ∼2.20 Å), O⋯H/H⋯O (18.4%; *d*_i_ + *d*_e_ ∼2.44 Å), C⋯H/H⋯C (18.0%; *d*_i_ + *d*_e_ ∼2.86 Å), S⋯H/H⋯S (8.2%; *d*_i_ + *d*_e_ ∼2.74 Å), Cl⋯H/H⋯Cl (7.2%; *d*_i_ + *d*_e_ ∼2.72 Å) and other contacts (14.0%). Almost all of these contacts are shorter than their corres­ponding sum van der Waals radii, with H⋯H, O⋯H, C⋯H, S⋯H and Cl⋯H being ∼2.4, ∼2.72, ∼2.9, ∼3.0 and ∼2.95 Å, respectively.

The DTBA and 2CBA mol­ecules display similar fingerprint patterns having a claw-like profile in the respective full fingerprint plots, implying the existence of nearly identical inter­actions between the mol­ecules which is expected considering the similarity of their mol­ecular structures. Detailed analysis of the decomposed fingerprint plots shows that H⋯H is the most prevalent contact for the mol­ecules, with the percentage contribution to the overall contacts of 29.7 and 25.0% and minimum *d*_i_ + *d*_e_ contact distance of ∼2.18 and ∼2.24 Å for DTBA and 2CBA, respectively. The O⋯H/H⋯O contacts are the second most dominant contact for the individual mol­ecules which lead to the distinctive spikes in the corresponding decomposed fingerprint plots with a contribution of 26.4% for DTBA and 22.2% for 2CBA. Further delin­eation of the contact shows that DTBA possesses about 11.1% of (inter­nal)-H⋯O-(external) and 15.3% (inter­nal)-O⋯H-(external) compared to 2CBA with 10.9 and 11.2% of the equivalent contacts, both with approximately the same *d*_i_ + *d*_e_ contact distance of ∼1.70 Å for DTBA and ∼1.62 Å for 2CBA. Additional contacts for DTBA and 2CBA are respectively dominated by C⋯H/H⋯C (17.5%, *d*_i_ + *d*_e_ ∼2.18 Å; 14.8%, *d*_i_ + *d*_e_ ∼3.16 Å), S⋯H/H⋯S (12.3%, *d*_i_ + *d*_e_ ∼2.72 Å; 1.5%, *d*_i_ + *d*_e_ ∼3.38 Å) and Cl⋯H/H⋯Cl (2.8%, *d*_i_ + *d*_e_ ∼2.74 Å; 17.7%, *d*_i_ + *d*_e_ ∼2.74 Å). As for the DMF solvent mol­ecule, this exhibits a relatively different claw-like profile with several disproportional spikes observed in the fingerprint plot mainly owing to the asymmetric inter­action environment for the O⋯H/ H⋯O contact, in which the contribution of (inter­nal)-O⋯H-(external) contact to the Hirshfeld surfaces is about 14.6% (*d*_i_ + *d*_e_ ∼1.60 Å), while the (inter­nal)-H⋯O-(external) contact is about 11.2% (*d*_i_ + *d*_e_ ∼2.22 Å) that can be summed up to yield an overall 25.8%. The contribution of other short contacts is noted in decreasing order: H⋯H (47.4%, *d*_i_ + *d*_e_ ∼2.20 Å), C⋯H/ H⋯C (15.4%, *d*_i_ + *d*_e_ ∼2.90 Å) and H⋯S (4.4%, *d*_i_ + *d*_e_ ∼3.36 Å), respectively.

## Computational chemistry study   

The energy calculations through *Crystal Explorer 17*, Table 2 ▸, indicate that the strongest inter­action occurs between the hydrogen-bonded DTBA and 2CBA mol­ecules [DTBA-O—H⋯O(carbonyl-2CBA)/DTBA=O⋯H—O-(hydroxyl-2CBA)] dimer with an inter­action energy (*E*_int_) of −73.2 kJ mol^−1^. This energy is about one and a half-fold greater than the second strongest inter­action that occurs between DTBA-DMF [DTBA-O—H⋯O(carbonyl-DMF)/ DTBA=O⋯H—C-(DMF)] with an *E*_int_ = −45.9 kJ mol^−1^. The disparity in energy is likely due the replacement of one O—H⋯O hydrogen bond with a C—H⋯O inter­action in the latter inter­action.

On the other hand, the π–π inter­action between the hydrogen bond-mediated dimer of (DTBA)_2_ and the 2CBA-benzene ring gives an energy of −15.9 kJ mol^−1^ which is considered weak in nature. This indicates the energy is mainly dominated by dispersive forces, Table 2 ▸, which validates the previous finding on ESP mapping. Inter­estingly, a recent study demonstrated that the presence of external agents such as Lewis acids may either increase or decrease the strength of resonance assisted hydrogen bonds (RAHB) depending on the position of inter­action of the external agent with a carb­oxy­lic acid dimer (Grabowski, 2008 ▸). The *E*_int_ for other inter­actions present in the crystal were also calculated and the results are summarized as in Table 2 ▸. Generally, the energies for these inter­actions range between −23.4 to −6.5 kJ mol^−1^ which can be considered weak.

**Figure 5:**
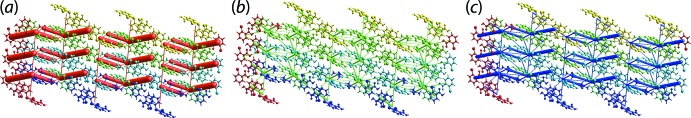
Energy framework of (I) as viewed down along the *b* axis, showing the energy framework comprising (*a*) electrostatic potential force, (*b*) dispersion force and (*c*) total energy. The cylindrical radii are proportional to the relative strength of the respective energies and they were scaled by a factor of 80 with a cut-off energy value of 5 kJ mol^−1^ within 4 × 4 × 4 unit cells.

The energy frameworks of (I) were also generated. The results of the calculations show that the mol­ecular packing is mainly governed by electrostatic forces which can be attributed to the strong O—H⋯O inter­actions, Fig. 5 ▸. The inter­actions coupled with the near orthogonal arrangement of the two carb­oxy­lic acid moieties of DTBA lead to a discrete, directional V-shape electrostatic energy topology which is arranged in an alternate array along the *c*-axis direction. A relatively weaker dispersion force co-exists along with the main energy framework due to π–π inter­actions which help to sustain the overall mol­ecular packing of (I).

A structural analogue of (I) in the literature is the 2:1 co-crystal composed of two DTBA mol­ecules and the isomeric 3-chloro­benzoic acid (3CBA) mol­ecule, (II) (Tan & Tiekink, 2019*b* ▸). Unlike (I), in which hydrogen bonds are formed between DTBA, 2CBA and DMF to result in a three-mol­ecule aggregate, Fig. 2 ▸(*a*), in (II) the two DTBA mol­ecules (DTBA-IIa and DTBA-IIb) form hydrogen bonds with each other, to yield a non-symmetric homosynthon, and with the two remaining carb­oxy­lic acid groups being hydrogen bonded to two 3CBA mol­ecules to give rise to a four-mol­ecule aggregate.

**Figure 6:**
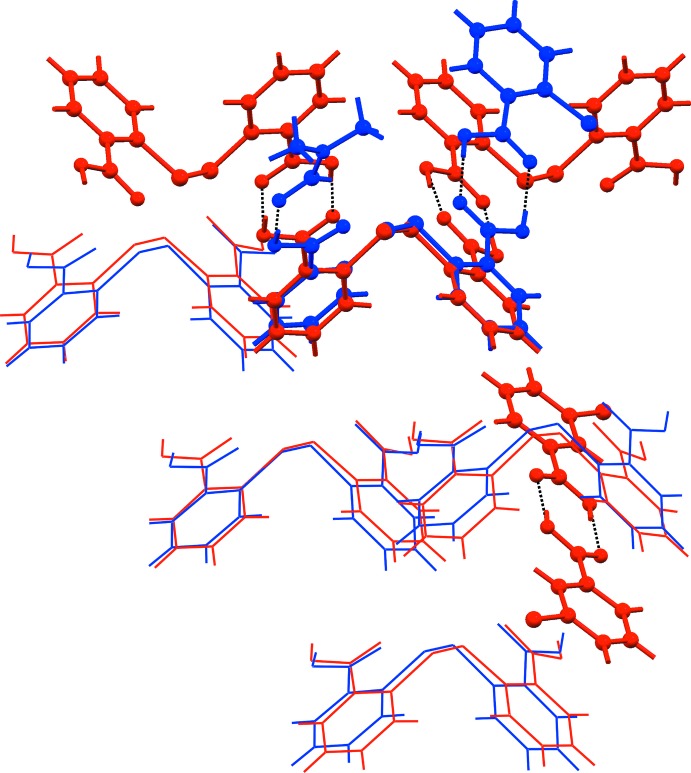
A comparison of the mol­ecular packing between (I) (blue) and (II) (red), showing the similarity between five pairs of DTBA mol­ecules with an overall r.m.s. deviation of 0.4 Å.

A mol­ecular cluster of (I) and (II) containing 20 mol­ecules was subjected to mol­ecular packing analysis using *Mercury* (Macrae *et al.*, 2006 ▸), with the geometric tolerances being set to the default values (20% for distance and 20° for angle tolerance); mol­ecular inversions were allowed during the comparison. The study shows that there are five pairs of DTBA mol­ecules from (I) and (II) which exhibit close similarity in the mol­ecular packing with an r.m.s. deviation of 0.4 Å, Fig. 6 ▸.

**Figure 7:**
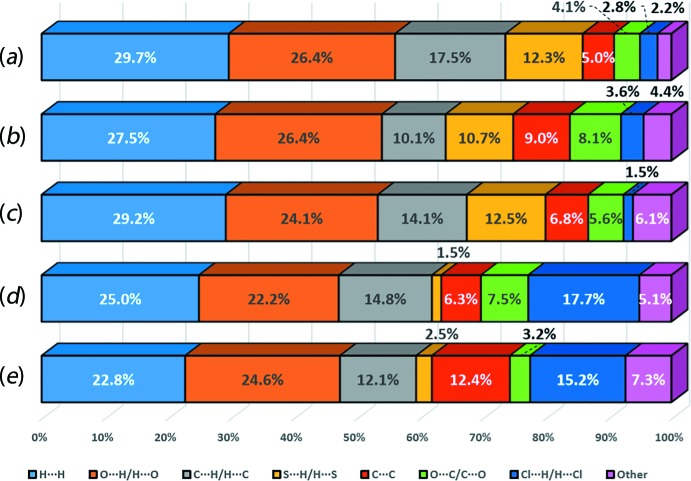
Percentage distribution of the corresponding close contacts to the Hirshfeld surfaces of (*a*) DTBA in (I), (*b*) first DTBA mol­ecule in (II), (*c*) second DTBA mol­ecule in (II), (*d*) 2CBA in (I) and (*e*) 3CBA in (II).

Both (I) and (II) also exhibit similarity in terms of their close contacts as evidenced from the percentage contribution of the corresponding contacts obtained through Hirshfeld surface analysis for the DTBA mol­ecules in (I) and (II), 2CBA in (I) or 3CBA in (II), Fig. 7 ▸. In general, the variations in contributions between those DTBA mol­ecules as well as 2CBA and 3CBA are relatively small: these differences range from 0.2 to 2.9% and 1.0 to 2.7% respectively. Exceptions are noted in the C⋯H/ H⋯C contacts which contribute about 17.5% of the overall contacts in DTBA-I, that is about 7.4 and 3.4% higher than the contacts in DTBA-IIa and DTBA-IIb, respectively. On the other hand, a relatively higher contribution is observed for the C⋯C contacts in 3CBA (*ca* 12.4%) which is approximately 6% greater than 2CBA in (I) (*ca* 6.3%).

## Database survey   

There are over 200 structures included in the Cambridge Structural Database (version5.40; Groom *et al.*, 2016 ▸) featuring hydrogen bonds between carb­oxy­lic acid residues and DMF. The most relevant structure is that of the 1:2 DTBA:DMF solvate (Cai *et al.*, 2006 ▸). Here, both carb­oxy­lic acid residues engage in hydrogen bonding inter­actions with DMF mol­ecules akin to that seen in (I). There are approximately 250 structures where (non-coordinated) DMF and a carb­oxy­lic acid residue are present in the same crystal but no hydrogen bonding is evident between them. This suggest a 40% likelihood of hydrogen bonding between carb­oxy­lic acids and DMF, a percentage higher than for the formation of the eight-membered {⋯HOCO}_2_ synthon in carb­oxy­lic acid structures, *i.e*. 33%, emphasizing that this particular synthon can be readily disrupted in the presence of competing synthons (Allen *et al.*, 1999 ▸).

## Synthesis and crystallization   

All chemical precursors were of reagent grade and used as received without further purification. 2-Mercapto­benzoic acid (Merck; 0.154 g, 0.001 mol) was mixed with 2-chloro­benzoic acid (Hopkin & Williams, 0.157 g, 0.001 mol) and ground for 15 mins in the presence of a few drops of methanol. The procedure was repeated three times. Colourless blocks were obtained through the careful layering of toluene (1 ml) on an *N*,*N*-di­methyl­formamide (1 ml) solution of the ground mixture. M.p. 437.3–438.9 K. IR (cm^−1^): 3076 *ν*(C—H), 1678 *ν*(C=O), 1473 *ν*(C=C), 1426 *δ*(C—H), 736 *δ*(C—Cl).

## Refinement   

Crystal data, data collection and structure refinement details are summarized in Table 3 ▸. The carbon-bound H atoms were placed in calculated positions (C—H = 0.93–0.96 Å) and were included in the refinement in the riding-model approximation, with *U*_iso_(H) set to 1.2–1.5*U*_eq_(C). The oxygen-bound H atoms were located from difference Fourier maps and refined without constraint. Owing to poor agreement, one reflection, *i.e*. (4 2 2), was omitted from the final cycles of refinement.

## Supplementary Material

Crystal structure: contains datablock(s) I, global. DOI: 10.1107/S205698901900375X/hb7808sup1.cif

Structure factors: contains datablock(s) I. DOI: 10.1107/S205698901900375X/hb7808Isup2.hkl

Supporting information file. DOI: 10.1107/S205698901900375X/hb7808Isup3.cml

CCDC reference: 1903993

Additional supporting information:  crystallographic information; 3D view; checkCIF report

## Figures and Tables

**Table 1:** Hydrogen-bond geometry (Å, °)

*D*—H⋯*A*	*D*—H	H⋯*A*	*D*⋯*A*	*D*—H⋯*A*
O2—H2*O*⋯O3	0.83 (6)	1.86 (7)	2.687 (4)	176 (9)
O4—H4*O*⋯O1	0.73 (7)	1.88 (6)	2.612 (4)	175 (5)
O6—H6*O*⋯O7	0.87 (5)	1.73 (5)	2.594 (4)	172 (5)
C3—H3⋯O4^i^	0.95	2.57	3.363 (5)	142
C10—H10⋯O2^ii^	0.95	2.54	3.331 (5)	141
C11—H11⋯S1^ii^	0.95	2.83	3.544 (4)	133
C22—H22⋯O5	0.95	2.33	3.095 (5)	138
C24—H24*B*⋯S2^iii^	0.98	2.83	3.531 (4)	129

Symmetry codes: (i) 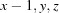; (ii) 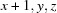; (iii) 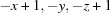.

**Table 2:** Inter­action energies (kJ mol^−1^) for selected close contacts

Contact	*E*_electrostatic_	*E*_polarization_	*E*_dispersion_	*E*_exchange-repulsion_	*E*_total_	Symmetry operation
O2—H2⋯O3/O4—H4*O*⋯O1	−123.7	−28.0	−13.0	145.1	−73.2	*x*, *y*, *z*
O6—H6*O*⋯O7/C22—H22⋯O5	−82.4	−19.2	−11.4	105.7	−45.9	*x*, *y*, *z*
*Cg*1(C9/C14)⋯*Cg*2(C2/C7)/C6—H16⋯Cl1	−4.1	−1.7	−41.9	30.3	−23.4	1 − *x*, 1 − *y*, − *z*
*Cg*3(C1O1O2⋯C8O3O4)⋯*Cg*2(C2/C7)	−1.0	−1.8	−30.7	21.6	−15.9	1 − *x*, − *y*, − *z*
C11—H11⋯S1/C11—H11⋯O3	−11.6	−2.2	−15.0	21.3	−13.8	−1 + *x*, *y*, *z*
C24—H24*C*⋯S2/C24—H24*C*⋯O5	−10.3	−2.5	−14.3	19.5	−13.2	1 − *x*, − *y*, 1 − *z*
C10—H10⋯O2/C3—H3⋯O4	−2.4	−1.1	−14.5	15.4	−6.5	−*x*, −*y*, 1 − *z*

**Table 3:** Experimental details

Crystal data	
Chemical formula	C_14_H_10_O_4_S_2_·C_7_H_5_ClO_2_·C_3_H_7_NO
*M*_r_	536.00
Crystal system, space group	Triclinic, *P* 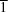
Temperature (K)	100
*a*, *b*, *c* (Å)	7.7487 (3), 7.8575 (3), 21.4486 (6)
α, β, γ (°)	86.136 (3), 88.693 (2), 65.080 (4)
*V* (Å^3^)	1181.61 (8)
*Z*	2
Radiation type	Cu *K*α
μ (mm^−1^)	3.50
Crystal size (mm)	0.19 × 0.12 × 0.03

Data collection	
Diffractometer	XtaLAB Synergy, Dualflex, AtlasS2
Absorption correction	Gaussian (*CrysAlis PRO*; Rigaku OD, 2018 ▸)
*T*_min_, *T*_max_	0.413, 1.000
No. of measured, independent and observed [*I* > 2σ(*I*)] reflections	40239, 4915, 4507
*R*_int_	0.057
(sin θ/λ)_max_ (Å^−1^)	0.630

Refinement	
*R*[*F* ^2^ > 2σ(*F* ^2^)], *wR*(*F* ^2^), *S*	0.067, 0.203, 1.10
No. of reflections	4915
No. of parameters	330
H-atom treatment	H atoms treated by a mixture of independent and constrained refinement
Δρ_max_, Δρ_min_ (e Å^−3^)	1.21, −0.83

Computer programs: *CrysAlis PRO* (Rigaku OD, 2018 ▸), *SHELXT* (Sheldrick, 2015*b* ▸), *SHELXL* (Sheldrick, 2015*a* ▸), *ORTEP-3 for Windows* (Farrugia, 2012 ▸), *OLEX2* (Dolomanov *et al.*, 2009 ▸) and *Mercury* (Macrae *et al.*, 2006 ▸) and *publCIF* (Westrip, 2010 ▸).

## supplementary crystallographic information

### Crystal data

**Table:**

C_14_H_10_O_4_S_2_·C_7_H_5_ClO_2_·C_3_H_7_NO	*Z* = 2
*M**_r_* = 536.00	*F*(000) = 556
Triclinic, *P*1	*D*_x_ = 1.506 Mg m^−^^3^
*a* = 7.7487 (3) Å	Cu *K*α radiation, λ = 1.54184 Å
*b* = 7.8575 (3) Å	Cell parameters from 21119 reflections
*c* = 21.4486 (6) Å	θ = 4.0–76.1°
α = 86.136 (3)°	µ = 3.50 mm^−^^1^
β = 88.693 (2)°	*T* = 100 K
γ = 65.080 (4)°	Plate, colourless
*V* = 1181.61 (8) Å^3^	0.19 × 0.12 × 0.03 mm

### Data collection

**Table:**

XtaLAB Synergy, Dualflex, AtlasS2 diffractometer	4915 independent reflections
Radiation source: micro-focus sealed X-ray tube, PhotonJet (Cu) X-ray Source	4507 reflections with *I* > 2σ(*I*)
Mirror monochromator	*R*_int_ = 0.057
Detector resolution: 5.2558 pixels mm^-1^	θ_max_ = 76.1°, θ_min_ = 4.1°
ω scans	*h* = −9→9
Absorption correction: gaussian (CrysAlisPro; Rigaku OD, 2018)	*k* = −9→9
*T*_min_ = 0.413, *T*_max_ = 1.000	*l* = −24→26
40239 measured reflections	

### Refinement

**Table:**

Refinement on *F*^2^	Primary atom site location: dual
Least-squares matrix: full	Hydrogen site location: mixed
*R*[*F*^2^ > 2σ(*F*^2^)] = 0.067	H atoms treated by a mixture of independent and constrained refinement
*wR*(*F*^2^) = 0.203	*w* = 1/[σ^2^(*F*_o_^2^) + (0.1187*P*)^2^ + 2.0348*P*] where *P* = (*F*_o_^2^ + 2*F*_c_^2^)/3
*S* = 1.10	(Δ/σ)_max_ = 0.001
4915 reflections	Δρ_max_ = 1.21 e Å^−^^3^
330 parameters	Δρ_min_ = −0.83 e Å^−^^3^
0 restraints	

### Special details

**Table:**

Geometry. All esds (except the esd in the dihedral angle between two l.s. planes) are estimated using the full covariance matrix. The cell esds are taken into account individually in the estimation of esds in distances, angles and torsion angles; correlations between esds in cell parameters are only used when they are defined by crystal symmetry. An approximate (isotropic) treatment of cell esds is used for estimating esds involving l.s. planes.

### Fractional atomic coordinates and isotropic or equivalent isotropic displacement parameters (Å^2^)

**Table:**

	*x*	*y*	*z*	*U*_iso_*/*U*_eq_	
S1	0.59149 (11)	0.53119 (12)	0.76852 (4)	0.0306 (2)	
Cl1	0.45069 (12)	0.95992 (13)	1.14356 (4)	0.0349 (2)	
S2	0.59937 (12)	0.53437 (12)	0.67272 (4)	0.0308 (2)	
O4	0.3171 (4)	0.6699 (4)	0.95268 (11)	0.0326 (5)	
H4O	0.364 (8)	0.709 (7)	0.973 (3)	0.052 (15)*	
O3	0.5740 (3)	0.5927 (3)	0.89192 (10)	0.0296 (5)	
O2	0.7735 (4)	0.6833 (4)	0.97404 (12)	0.0354 (6)	
H2O	0.712 (8)	0.652 (8)	0.950 (3)	0.060 (16)*	
O1	0.5007 (3)	0.7890 (4)	1.02638 (11)	0.0344 (6)	
O5	0.6630 (4)	0.5440 (4)	0.55037 (12)	0.0418 (6)	
O6	0.8588 (4)	0.3049 (4)	0.49649 (12)	0.0410 (6)	
H6O	0.841 (7)	0.395 (7)	0.468 (2)	0.045 (13)*	
O7	0.8389 (4)	0.5600 (4)	0.41008 (12)	0.0434 (6)	
N1	0.7396 (5)	0.8751 (5)	0.39798 (14)	0.0400 (7)	
C9	0.2951 (5)	0.5828 (4)	0.85135 (15)	0.0267 (6)	
C1	0.6657 (5)	0.7680 (5)	1.02109 (15)	0.0275 (6)	
C14	0.3607 (5)	0.5473 (5)	0.78952 (15)	0.0270 (6)	
C8	0.4089 (4)	0.6141 (5)	0.90013 (15)	0.0267 (6)	
C2	0.7656 (5)	0.8312 (5)	1.06648 (15)	0.0280 (6)	
C12	0.0633 (5)	0.5400 (5)	0.76228 (16)	0.0306 (7)	
H12	−0.016266	0.526211	0.731834	0.037*	
C10	0.1132 (5)	0.5965 (5)	0.86691 (15)	0.0291 (7)	
H10	0.068160	0.622104	0.908335	0.035*	
C13	0.2422 (5)	0.5262 (5)	0.74536 (15)	0.0295 (7)	
H13	0.284561	0.502362	0.703600	0.035*	
C6	0.7871 (5)	0.9609 (5)	1.16394 (16)	0.0323 (7)	
H6A	0.730171	1.015228	1.201643	0.039*	
C11	−0.0011 (5)	0.5736 (5)	0.82314 (16)	0.0318 (7)	
H11	−0.123116	0.580647	0.834470	0.038*	
C5	0.9736 (5)	0.9304 (5)	1.15083 (17)	0.0345 (7)	
H5	1.044152	0.963015	1.179689	0.041*	
C7	0.6831 (5)	0.9126 (5)	1.12226 (15)	0.0274 (6)	
C16	0.6785 (5)	0.1584 (5)	0.70069 (16)	0.0338 (7)	
H16	0.626413	0.196047	0.740635	0.041*	
C15	0.6826 (5)	0.2925 (5)	0.65578 (16)	0.0312 (7)	
C21	0.7543 (5)	0.3771 (5)	0.54597 (16)	0.0336 (7)	
C20	0.7578 (5)	0.2356 (5)	0.59629 (16)	0.0330 (7)	
C3	0.9540 (5)	0.8034 (5)	1.05434 (17)	0.0319 (7)	
H3	1.012239	0.749575	1.016708	0.038*	
C22	0.7667 (6)	0.7194 (5)	0.42990 (18)	0.0413 (9)	
H22	0.727643	0.728713	0.472319	0.050*	
C17	0.7494 (6)	−0.0294 (5)	0.68806 (18)	0.0402 (8)	
H17	0.743500	−0.119055	0.719045	0.048*	
C19	0.8321 (6)	0.0448 (5)	0.58490 (18)	0.0398 (8)	
H19	0.885722	0.005247	0.545262	0.048*	
C4	1.0573 (5)	0.8524 (5)	1.09581 (18)	0.0368 (8)	
H4A	1.184608	0.832653	1.086567	0.044*	
C18	0.8290 (6)	−0.0872 (5)	0.63029 (19)	0.0432 (9)	
H18	0.880856	−0.216500	0.622025	0.052*	
C23	0.7881 (7)	0.8803 (6)	0.33196 (18)	0.0441 (9)	
H23A	0.854211	0.962019	0.324500	0.066*	
H23B	0.671435	0.929459	0.306655	0.066*	
H23C	0.871087	0.752967	0.320389	0.066*	
C24	0.6454 (7)	1.0572 (6)	0.4254 (2)	0.0487 (10)	
H24A	0.626207	1.037588	0.470118	0.073*	
H24B	0.521838	1.129721	0.404825	0.073*	
H24C	0.724883	1.126361	0.419463	0.073*	

### Atomic displacement parameters (Å^2^)

**Table:**

	*U*^11^	*U*^22^	*U*^33^	*U*^12^	*U*^13^	*U*^23^
S1	0.0263 (4)	0.0454 (5)	0.0243 (4)	−0.0182 (3)	0.0065 (3)	−0.0111 (3)
Cl1	0.0324 (4)	0.0482 (5)	0.0290 (4)	−0.0208 (4)	0.0096 (3)	−0.0119 (3)
S2	0.0342 (4)	0.0354 (4)	0.0240 (4)	−0.0152 (3)	0.0080 (3)	−0.0083 (3)
O4	0.0306 (12)	0.0483 (14)	0.0260 (12)	−0.0220 (11)	0.0055 (9)	−0.0132 (10)
O3	0.0229 (11)	0.0420 (13)	0.0266 (11)	−0.0153 (10)	0.0053 (8)	−0.0106 (9)
O2	0.0300 (12)	0.0515 (15)	0.0287 (12)	−0.0195 (11)	0.0069 (10)	−0.0152 (11)
O1	0.0266 (12)	0.0495 (15)	0.0309 (12)	−0.0180 (11)	0.0065 (9)	−0.0158 (10)
O5	0.0530 (16)	0.0358 (14)	0.0291 (13)	−0.0115 (12)	0.0136 (11)	−0.0056 (10)
O6	0.0519 (16)	0.0363 (13)	0.0284 (13)	−0.0123 (12)	0.0155 (11)	−0.0069 (11)
O7	0.0574 (17)	0.0385 (14)	0.0313 (13)	−0.0169 (12)	0.0120 (12)	−0.0081 (11)
N1	0.0502 (19)	0.0412 (17)	0.0297 (15)	−0.0200 (15)	0.0053 (13)	−0.0058 (13)
C9	0.0266 (15)	0.0296 (15)	0.0242 (15)	−0.0117 (12)	0.0016 (12)	−0.0056 (12)
C1	0.0259 (15)	0.0313 (16)	0.0251 (15)	−0.0113 (12)	0.0035 (12)	−0.0059 (12)
C14	0.0255 (15)	0.0305 (15)	0.0267 (15)	−0.0127 (12)	0.0046 (12)	−0.0077 (12)
C8	0.0252 (15)	0.0316 (16)	0.0250 (15)	−0.0131 (12)	0.0034 (12)	−0.0067 (12)
C2	0.0254 (15)	0.0316 (16)	0.0257 (15)	−0.0105 (12)	0.0019 (12)	−0.0040 (12)
C12	0.0279 (16)	0.0355 (17)	0.0302 (16)	−0.0141 (13)	−0.0001 (13)	−0.0082 (13)
C10	0.0280 (16)	0.0349 (17)	0.0262 (16)	−0.0143 (13)	0.0054 (12)	−0.0082 (13)
C13	0.0278 (16)	0.0372 (17)	0.0242 (15)	−0.0136 (13)	0.0031 (12)	−0.0080 (13)
C6	0.0353 (18)	0.0357 (17)	0.0248 (16)	−0.0134 (14)	−0.0001 (13)	−0.0042 (13)
C11	0.0255 (16)	0.0386 (18)	0.0332 (17)	−0.0145 (14)	0.0034 (13)	−0.0097 (14)
C5	0.0334 (18)	0.0379 (18)	0.0333 (18)	−0.0152 (14)	−0.0034 (14)	−0.0062 (14)
C7	0.0269 (15)	0.0302 (15)	0.0250 (15)	−0.0118 (12)	0.0033 (12)	−0.0042 (12)
C16	0.0318 (17)	0.0385 (18)	0.0274 (16)	−0.0109 (14)	0.0060 (13)	−0.0057 (13)
C15	0.0268 (16)	0.0368 (17)	0.0273 (16)	−0.0102 (13)	0.0042 (12)	−0.0076 (13)
C21	0.0359 (18)	0.0390 (18)	0.0255 (16)	−0.0146 (15)	0.0080 (13)	−0.0095 (13)
C20	0.0335 (17)	0.0372 (18)	0.0264 (16)	−0.0125 (14)	0.0052 (13)	−0.0062 (13)
C3	0.0259 (16)	0.0387 (18)	0.0331 (17)	−0.0142 (14)	0.0042 (13)	−0.0125 (14)
C22	0.046 (2)	0.040 (2)	0.0353 (19)	−0.0148 (17)	0.0088 (16)	−0.0088 (15)
C17	0.043 (2)	0.0355 (18)	0.0367 (19)	−0.0116 (16)	0.0097 (15)	−0.0009 (15)
C19	0.044 (2)	0.0384 (19)	0.0321 (18)	−0.0115 (16)	0.0110 (15)	−0.0099 (15)
C4	0.0259 (16)	0.046 (2)	0.0392 (19)	−0.0151 (15)	0.0014 (14)	−0.0108 (16)
C18	0.052 (2)	0.0323 (18)	0.040 (2)	−0.0119 (16)	0.0109 (17)	−0.0082 (15)
C23	0.060 (3)	0.048 (2)	0.0304 (18)	−0.028 (2)	0.0074 (17)	−0.0047 (16)
C24	0.061 (3)	0.038 (2)	0.041 (2)	−0.0153 (19)	0.0057 (19)	−0.0063 (17)

### Geometric parameters (Å, º)

**Table:**

S1—S2	2.0531 (11)	C13—H13	0.9500
S1—C14	1.788 (3)	C6—H6A	0.9500
Cl1—C7	1.736 (3)	C6—C5	1.387 (5)
S2—C15	1.791 (4)	C6—C7	1.389 (5)
O4—H4O	0.74 (6)	C11—H11	0.9500
O4—C8	1.317 (4)	C5—H5	0.9500
O3—C8	1.229 (4)	C5—C4	1.384 (5)
O2—H2O	0.82 (6)	C16—H16	0.9500
O2—C1	1.320 (4)	C16—C15	1.389 (5)
O1—C1	1.222 (4)	C16—C17	1.384 (5)
O5—C21	1.209 (4)	C15—C20	1.411 (5)
O6—H6O	0.87 (5)	C21—C20	1.488 (5)
O6—C21	1.326 (4)	C20—C19	1.398 (5)
O7—C22	1.238 (5)	C3—H3	0.9500
N1—C22	1.298 (5)	C3—C4	1.385 (5)
N1—C23	1.459 (5)	C22—H22	0.9500
N1—C24	1.463 (5)	C17—H17	0.9500
C9—C14	1.412 (4)	C17—C18	1.388 (5)
C9—C8	1.480 (4)	C19—H19	0.9500
C9—C10	1.402 (5)	C19—C18	1.381 (6)
C1—C2	1.489 (5)	C4—H4A	0.9500
C14—C13	1.398 (5)	C18—H18	0.9500
C2—C7	1.404 (4)	C23—H23A	0.9800
C2—C3	1.403 (5)	C23—H23B	0.9800
C12—H12	0.9500	C23—H23C	0.9800
C12—C13	1.386 (5)	C24—H24A	0.9800
C12—C11	1.389 (5)	C24—H24B	0.9800
C10—H10	0.9500	C24—H24C	0.9800
C10—C11	1.376 (5)		
			
C14—S1—S2	105.47 (11)	C6—C7—C2	120.5 (3)
C15—S2—S1	104.62 (12)	C15—C16—H16	119.5
C8—O4—H4O	113 (4)	C17—C16—H16	119.5
C1—O2—H2O	110 (4)	C17—C16—C15	120.9 (3)
C21—O6—H6O	109 (3)	C16—C15—S2	121.2 (3)
C22—N1—C23	122.3 (3)	C16—C15—C20	119.1 (3)
C22—N1—C24	121.4 (3)	C20—C15—S2	119.7 (3)
C23—N1—C24	116.1 (3)	O5—C21—O6	123.2 (3)
C14—C9—C8	122.3 (3)	O5—C21—C20	122.2 (3)
C10—C9—C14	119.3 (3)	O6—C21—C20	114.6 (3)
C10—C9—C8	118.4 (3)	C15—C20—C21	120.5 (3)
O2—C1—C2	113.6 (3)	C19—C20—C15	119.0 (3)
O1—C1—O2	122.2 (3)	C19—C20—C21	120.5 (3)
O1—C1—C2	124.2 (3)	C2—C3—H3	119.2
C9—C14—S1	120.0 (2)	C4—C3—C2	121.7 (3)
C13—C14—S1	120.9 (2)	C4—C3—H3	119.2
C13—C14—C9	119.0 (3)	O7—C22—N1	125.9 (4)
O4—C8—C9	114.4 (3)	O7—C22—H22	117.0
O3—C8—O4	122.8 (3)	N1—C22—H22	117.0
O3—C8—C9	122.8 (3)	C16—C17—H17	119.9
C7—C2—C1	123.4 (3)	C16—C17—C18	120.2 (3)
C3—C2—C1	118.8 (3)	C18—C17—H17	119.9
C3—C2—C7	117.8 (3)	C20—C19—H19	119.4
C13—C12—H12	119.6	C18—C19—C20	121.1 (3)
C13—C12—C11	120.9 (3)	C18—C19—H19	119.4
C11—C12—H12	119.6	C5—C4—C3	119.5 (3)
C9—C10—H10	119.5	C5—C4—H4A	120.3
C11—C10—C9	121.1 (3)	C3—C4—H4A	120.3
C11—C10—H10	119.5	C17—C18—H18	120.2
C14—C13—H13	119.9	C19—C18—C17	119.5 (4)
C12—C13—C14	120.3 (3)	C19—C18—H18	120.2
C12—C13—H13	119.9	N1—C23—H23A	109.5
C5—C6—H6A	119.8	N1—C23—H23B	109.5
C5—C6—C7	120.4 (3)	N1—C23—H23C	109.5
C7—C6—H6A	119.8	H23A—C23—H23B	109.5
C12—C11—H11	120.3	H23A—C23—H23C	109.5
C10—C11—C12	119.4 (3)	H23B—C23—H23C	109.5
C10—C11—H11	120.3	N1—C24—H24A	109.5
C6—C5—H5	119.9	N1—C24—H24B	109.5
C4—C5—C6	120.2 (3)	N1—C24—H24C	109.5
C4—C5—H5	119.9	H24A—C24—H24B	109.5
C2—C7—Cl1	123.5 (3)	H24A—C24—H24C	109.5
C6—C7—Cl1	116.0 (3)	H24B—C24—H24C	109.5
			
S1—S2—C15—C16	17.0 (3)	C2—C3—C4—C5	0.2 (6)
S1—S2—C15—C20	−161.5 (3)	C10—C9—C14—S1	179.5 (3)
S1—C14—C13—C12	−179.7 (3)	C10—C9—C14—C13	−0.2 (5)
S2—S1—C14—C9	−168.5 (2)	C10—C9—C8—O4	−6.1 (4)
S2—S1—C14—C13	11.1 (3)	C10—C9—C8—O3	175.0 (3)
S2—C15—C20—C21	−4.9 (5)	C13—C12—C11—C10	1.1 (5)
S2—C15—C20—C19	176.4 (3)	C6—C5—C4—C3	−0.7 (6)
O2—C1—C2—C7	−175.4 (3)	C11—C12—C13—C14	−0.4 (5)
O2—C1—C2—C3	2.5 (5)	C5—C6—C7—Cl1	−179.4 (3)
O1—C1—C2—C7	3.7 (5)	C5—C6—C7—C2	0.3 (5)
O1—C1—C2—C3	−178.3 (3)	C7—C2—C3—C4	0.5 (5)
O5—C21—C20—C15	−11.7 (6)	C7—C6—C5—C4	0.4 (6)
O5—C21—C20—C19	167.0 (4)	C16—C15—C20—C21	176.5 (3)
O6—C21—C20—C15	168.3 (3)	C16—C15—C20—C19	−2.1 (5)
O6—C21—C20—C19	−13.0 (5)	C16—C17—C18—C19	−1.8 (7)
C9—C14—C13—C12	0.0 (5)	C15—C16—C17—C18	1.1 (6)
C9—C10—C11—C12	−1.3 (5)	C15—C20—C19—C18	1.5 (6)
C1—C2—C7—Cl1	−3.2 (5)	C21—C20—C19—C18	−177.2 (4)
C1—C2—C7—C6	177.1 (3)	C20—C19—C18—C17	0.5 (7)
C1—C2—C3—C4	−177.5 (3)	C3—C2—C7—Cl1	178.9 (3)
C14—C9—C8—O4	171.4 (3)	C3—C2—C7—C6	−0.8 (5)
C14—C9—C8—O3	−7.5 (5)	C17—C16—C15—S2	−177.6 (3)
C14—C9—C10—C11	0.8 (5)	C17—C16—C15—C20	0.9 (5)
C8—C9—C14—S1	2.1 (4)	C23—N1—C22—O7	1.9 (7)
C8—C9—C14—C13	−177.6 (3)	C24—N1—C22—O7	177.4 (4)
C8—C9—C10—C11	178.4 (3)		

### Hydrogen-bond geometry (Å, º)

**Table:**

*D*—H···*A*	*D*—H	H···*A*	*D*···*A*	*D*—H···*A*
O2—H2*O*···O3	0.83 (6)	1.86 (7)	2.687 (4)	176 (9)
O4—H4*O*···O1	0.73 (7)	1.88 (6)	2.612 (4)	175 (5)
O6—H6*O*···O7	0.87 (5)	1.73 (5)	2.594 (4)	172 (5)
C3—H3···O4^i^	0.95	2.57	3.363 (5)	142
C10—H10···O2^ii^	0.95	2.54	3.331 (5)	141
C11—H11···S1^ii^	0.95	2.83	3.544 (4)	133
C22—H22···O5	0.95	2.33	3.095 (5)	138
C24—H24*B*···S2^iii^	0.98	2.83	3.531 (4)	129

Symmetry codes: (i) *x*−1, *y*, *z*; (ii) *x*+1, *y*, *z*; (iii) −*x*+1, −*y*, −*z*+1.
